# Extracting Teeth

**Published:** 1898-02

**Authors:** F. C. M'Pherson


ARTICLE III.

### EXTRACTING TEETH.

BY DR. F. C. M'PHERSON, '97.

Read before the Alumni Association, December, 7th, 1897.

The operation of extracting decayed and painful teeth has been performed, in some manner, from very remote antiquity. At first it was with very unsuitable instruments, and in the roughest and most barbarous manner. In times past, and not even very remote, extracting teeth, bleeding, cupping, leeching, and indeed most other surgical operations, were consigned to the care of barbers, as not worthy the attention of learned physicians.

As this operation is more frequently performed than any or all other surgical operations combined, and as it is usually accompanied with more dread than any other operation of equal importance, and when unskillfully performed is a very painful one, sometimes even a dangerous one; it is therefore important that it should always be done in the most careful and skillful manner. Proper instruments should be selected, and used with the least exertion and force by the operator, and with the least possible injury to the surrounding parts.

The teeth of all the higher orders of animals are held in place by that form of attachment called gomphosis, which consists in insertion of the roots into sockets in the jaw. The roots are invested and the sockets lined with a dense, firm membrane, the pericementum. Strong fibers of this membrane penetrate the tooth cementum on one side, and the bone on the other, and it is necessary to tear these fibers loose from either one or the other, before the tooth can be extracted.

Sometimes the attachments are the strongest to the tooth, and in that case the pericementum will be brought out on extraction, attached to the tooth. Usually, the membrane is more closely attached to the walls of the boney sockets, and the fibers are torn out of the tooth, leaving the pericementum in the socket. The tooth is also sometimes retained by its shape, or by curves, or by enlargement of the root, and so firm is its attachment to the jaw in many instances, that it is only with extreme difficulty that it can be dislodged.

A few of these facts, perhaps, will remind us how important it is to consider all cases well before operating. It is a fact that many teeth are extracted that might and should be saved. Sometimes this is the fault of the dentist, and sometimes the patient insists upon the removal of teeth which should not be lost. It is the dentist's duty to instruct the patient in this respect, and to refuse to take out teeth which would better be left in. When necessary to extract, study the case well, find out the condition of the tooth to be extracted, and that of the surrounding parts. Study the form of the alveolar process around the tooth to be extracted, as it varies in thickness and shape according to the size of the tooth, and its arrangement in the arch. It is especially necessary to be familiar with the condition of the lower jaw at the second lower molar. The rising of the external oblique ridge for the formation of the anterior border of the coronoid process, causes a thickening of the buccal bony covering of the root, while at the third molar this ridge rises to a level with the border of the alveolar process, making the bony covering on the buccal side of the root about one-fourth of an inch thick.

This is of importance with reference to the extraction of the roots of this tooth, which in many cases is the most difficult to remove. In fact the second and third molars of the lower jaw are fixed in alveoli, hollowed out in the lingual side of the body of the bone, rather than in a process or ridge on the bone, as with teeth anterior. Caution should be used in every case when extracting to avoid unnecessary destruction of the alveolar process. Especial care should be used with the peridental membrane in extracting for old people. As age advances the peridental membrane becomes thinner, and the movements of the teeth are more restricted. In some cases the roots adhere to the alveolar process. It is well as a precation when extracting, to compress the gum around the roots of the tooth to be extracted with the thumb and index finger of the left hand, which will enable the operator to tell when the roots are loose from the process. Then giving the forceps a rotary motion before pulling, will materially lessen the danger of part of the process coming with the tooth. The greatest difficulty in extracting is usually found in the case of impacted teeth which lie horizontally instead of vertically in the jaw. This most frequently happens with the wisdom, or third molar teeth. They often develop just at the border of the ramus, so that there is not sufficient room for them. Sometimes they cause so much imflammation that the muscles become affected, and it is impossible to get the mouth sufficiently open for extraction. In these cases the first thing is to control this until the tooth can be reached. It may be necessary to give an anæthetic, and chip away the process until it can be taken out. Usually the Physic forcep will be a great help in these cases, if the second molar is in position. The blades are placed between the teeth, and the second molar being used as a fulcrum, the wisdom tooth is pried up and tipped over backward.

Teeth should be extracted with moderation in all cases. It is much easier and more effectual to accomplish this if the operator will use his body as a fulcrum, his arm being the lever, thereby lifting the tooth out slowly, thus avoiding the danger of injuring the surrounding parts, as well as the possible crushing of the tooth, which is often the case when the swinging arm motion is used. This has to be done more quickly in order to get the required power.

There are many methods employed to make extracting painless. The local application, hypodermically, of cocaine in some form is quite general, with very good success, providing it is properly applied.

From four to six injections in the gum around the neck of the tooth to be extracted are generally sufficient to get complete anæsthesia. The free margin of the gum should then be lanced, and if there is still a little sensation, insert a little more of the anæsthetic and then extract. If properly executed, 90 per cent. of all teeth can be extracted with satisfaction to the patient.

A local anæsthetic should never be employed unless one knows what it contains. No one can use any remedy safely unless he knows what it is. He cannot tell how much it will be safe to give. He must adapt his dose to the physical condition of the patient. There is no danger of the gums sloughing if the proper anæsthetic is employed. All instruments should be kept well sterilized, and the mouth should be sprayed with some good antiseptic after the operation.

But even with the greatest care accidents will sometimes happen, such as the breaking of a tooth, or of the process. When this occurs, the dentist should remember that the first thing is to stop any great flow of blood, and to compress the parts together.

Above all things he must not get excited or lose command of his faculties, remembering that unless there is a very grave injury there is little danger of life.—*The Dental Practitioner and Advertiser.*

